# *Bifidobacterium breve* CBT BR3 and *Lactiplantibacillus plantarum* CBT LP3 Alleviate Hepatic Steatosis by Suppressing Hepatic Lipogenesis and Improving Metabolic Hormone Profiles *In Vitro* and *In Vivo*

**DOI:** 10.4014/jmb.2604.04074

**Published:** 2026-08-12

**Authors:** Yeongju Yeo, Jong Won Kim, Yusook Chung, Gyeyeong Kong, Misun Yun, Sanghyun Lim

**Affiliations:** 1 R&D Center, Cell Biotech Co. Ltd., Gimpo 10003, Republic of Korea; 2 Advanced Convergence Research Division, World Institute of Kimchi, Gwangju 61755, Republic of Korea

**Keywords:** Hepatic steatosis, Lipogenesis, Probiotics, *Bifidobacterium breve*, *Lactiplantibacillus plantarum*

## Abstract

Hepatic steatosis is an early key event in metabolic dysfunction-associated steatotic liver disease, for which safe and effective interventions for reducing hepatic lipid accumulation are needed. To this end, we investigated the anti-steatotic effect of combining probiotics *Bifidobacterium breve* BR3 and *Lactiplantibacillus plantarum* LP3 (BR3LP3) on an *in vitro* oleic acid (OA)-treated human hepatocellular carcinoma (HepG2) cell model and an *in vivo* high-fat diet (HFD)-induced obese mouse model. In the OA-treated HepG2 cell model, BR3LP3 reduced OA-induced lipid accumulation without exhibiting cytotoxicity and modulated lipogenesis-related protein expression, while in the HFD-fed obese mouse model, oral BR3LP3 supplementation reduced body weight, fat mass, liver weight, and epididymal adipose tissue weight and improved several serum lipid, liver injury-related, and metabolic hormone parameters without affecting food intake. Moreover, when comparing BR3LP3-supplemented mice with those fed the HFD only, the former revealed reduced hepatic lipid droplet accumulation via histological analysis, while the expression levels of sterol regulatory element-binding protein-1c (*Srebp1c*), peroxisome proliferator-activated receptor γ (*Pparγ*), CCAAT/enhancer-binding protein alpha (*Cebpα*), acetyl-CoA carboxylase (*Acc*), and fatty acid synthase (*Fas*) were lower. Hence, our findings indicate that BR3LP3 supplementation attenuates hepatic lipid accumulation and the expression of hepatic lipogenesis-related markers while improving adiposity and systemic metabolic parameters. This suggests that BR3LP3 supplementation is a promising probiotic dietary strategy for improving hepatic steatosis and related metabolic dysfunction.

## Introduction

Hepatic steatosis is an early and pivotal manifestation of metabolic dysfunction-associated steatotic liver disease (MASLD), formerly known as nonalcoholic fatty liver disease. Hepatic steatosis is closely associated with obesity, insulin resistance, dyslipidemia, and progressive liver injury. Although steatosis might initially appear to be clinically silent, persistent hepatic lipid accumulation increases the risk for progression to steatohepatitis, fibrosis, and cirrhosis, along with broad cardiometabolic complications. Along with increasing rates of obesity and type 2 diabetes, the global burden of MASLD continues to increase, necessitating safe and effective interventions that can prevent excessive hepatic lipid deposition before it can advance to more severe liver disease [1-3].

A major driver of hepatic steatosis is dysregulated de novo lipogenesis. In the steatotic liver, lipogenic transcriptional programs involving sterol regulatory element-binding protein-1c (SREBP-1c), peroxisome proliferator-activated receptor γ (PPARγ), and CCAAT/enhancer-binding protein α (C/EBPα) enhance the expression of downstream enzymes such as acetyl-CoA carboxylase (ACC) and fatty acid synthase (FAS), which promote triglyceride accumulation and fatty acid synthesis, respectively. Because of their close association with hepatic lipid deposition, these transcription factors and enzymes are widely used as representative markers of hepatic lipogenesis and steatosis [4-8].

Given the close functional connection between the gut and the liver, dietary probiotics have been identified as promising candidates for improving hepatic steatosis and associated metabolic abnormalities. Recent systematic reviews and meta-analyses have shown that probiotic-based interventions can improve liver enzyme levels, lipid parameters, glycemic control, and inflammatory indices in patients with fatty liver disease, with multi-strain formulations often demonstrating greater efficacy than single-strain preparations [9-12]. Among the multi-strain formulations, combining *Bifidobacterium breve* BR3 and *Lactiplantibacillus plantarum* LP3 (BR3LP3) has shown promising metabolic benefits in animal studies. In mice, BR3LP3 has been reported to ameliorate high-fat diet (HFD)-induced metabolic disturbances, including reductions in body weight, adiposity, serum transaminase levels, and hepatic lipid accumulation [13]. Moreover, BR3LP3 supplementation has also been shown to improve body weight-related parameters and the gut microbiota composition in overweight dogs, which indicates the broad metabolic relevance of this formulation [14]. Furthermore, both strains reportedly have favorable safety, stability, and metabolic activity profiles [14, 15], thereby highlighting the potential of this combination as a probiotic for metabolic health improvement.

Previous work has demonstrated that BR3LP3 attenuates HFD-induced obesity and reduces body weight, adiposity, serum transaminase levels, and hepatic lipid accumulation, together with changing adipose tissue lipid metabolism, intestinal barrier-related markers, and gut microbiota composition [13]. However, the authors evaluated hepatic lipid accumulation as part of a broad systemic anti-obesity phenotype rather than principally investigating the responses of hepatocyte-like cells and liver-specific lipogenesis-related markers. To overcome this research gap, we evaluated the anti-steatotic potential of BR3LP3 in oleic acid (OA)-treated HepG2 cells and in a mouse model of HFD-induced obesity. We further investigated whether BR3LP3 modulated lipogenesis-related markers by assessing lipid accumulation, lipogenic protein expression, body composition, serum biochemical parameters, liver histology, and hepatic expression of various lipogenesis-related genes.

## Materials and Methods

### The Probiotic Supplement

The probiotic preparation BR3LP3 consisted of *B.breve* CBT BR3 (KCTC12201BP) and *L.plantarum* CBT LP3 (KCTC10782BP) (Cell Biotech (CBT); Republic of Korea).

For the *in vitro* experiments, supernatants from BR3 and LP3 cultures were used to evaluate whether soluble bacterial components released by BR3 and/or LP3 could influence lipid accumulation and lipogenesis-related protein expression in human hepatocellular carcinoma (HepG2) cells. To this end, the bacterial supernatants were applied to HepG2 cells at a final concentration of 500 or 1,000 μg/mL, as indicated for each experiment. For the *in vivo* experiments with mice, the probiotic mixture was suspended in 200 μL Dulbecco’s phosphate-buffered saline and administered through oral gavage once daily at a dose of 1.75 × 10^8^ (low dose) or 1.75 × 10^9^ (high dose) colony-forming unit (CFU)/day.

### Cell Culture and Treatment

The HepG2 cells, obtained from the American Type Culture Collection (ATCC, USA), were cultured in Dulbecco’s modified Eagle’s medium (Gibco, USA) supplemented with 10% fetal bovine serum (Gibco) and 1% penicillin–streptomycin (Gibco). Cells were maintained at 37°C in a humidified incubator containing 5% CO_2_ and subcultured to 70–80% confluence.

For lipid accumulation experiments, HepG2 cells were seeded at densities appropriate for each assay and allowed to attach for 24 h. They were then pretreated with test samples at final concentrations of 500 or 1,000 μg/mL for 4 h. Steatosis was induced by adding 1 mM OA, and the cells were incubated for an additional 24 h with or without test samples, while control cells were treated with the vehicle only.

### Cell Viability Assay

Cell viability was evaluated using Cell Counting Kit-8 (CCK-8; Dojindo Laboratories, Japan). To this end, HepG2 cells were seeded at a density of 5 × 10^3^ cells per well in 96-well plates and incubated for 24 h to allow attachment. They were then treated with test samples at the indicated concentrations in the presence or absence of 1 mM OA for 24 h. Afterward, 10 μL of CCK-8 solution was added to each well, followed by incubation for 1–2 h at 37°C. Subsequently, absorbance was measured at 450 nm using a SpectraMax ABS Plus microplate reader (Molecular Devices, USA). Cell viability is expressed as a percentage relative to the untreated control group. All experiments were performed in triplicate.

### Oil Red O Staining and Intracellular Lipid Quantification

Intracellular lipid accumulation was assessed using Oil Red O staining [16]. HepG2 cells were seeded in 6-well plates at a density of 3 × 10^5^ cells per well and treated with test samples and OA, as described above. Afterward, the cells were washed twice with phosphate-buffered saline (PBS) and fixed with 10% formalin for 30 min at room temperature. Following this, cells were washed with distilled water and stained with freshly prepared Oil Red O working solution for 15 min at room temperature. Excess stain was removed by washing several times with distilled water until the background was clear. Subsequently, lipid droplets were visualized under a light microscope (Olympus CKX41, Japan)

For quantitative analysis, lipid-bound dye was eluted with 100% isopropanol, after which the extracted dye was transferred to a 96-well plate and the absorbance was measured at 500 nm using a SpectraMax ABS Plus microplate reader. All experiments were performed in triplicate, and the results are expressed as relative lipid accumulation compared with the control group.

### Western Blot Analysis

Total protein was extracted from HepG2 cells using radioimmunoprecipitation assay lysis buffer (ELPIS Biotech, Republic of Korea) supplemented with a protease inhibitor cocktail (Roche, Switzerland). The total protein concentration was determined using the bicinchoninic acid assay (Thermo Fisher Scientific, USA). Equal amounts of protein (30 μg) were resolved on 8% or 10% sodium dodecyl sulfate-polyacrylamide gels and transferred onto polyvinylidenedifluoride membranes (Cytiva, Republic of Korea). The membranes were blocked with 5% skimmed milk and incubated overnight at 4°C with primary antibodies against PPARγ (Cat. No. 2435S), SREBP-1 (Cat. No. 95879S), C/EBPα (Cat. No. 8178S), FAS (Cat.No. 4233S), and β-Actin (Cat.No. 4970S) (all from Cell Signaling Technology, USA). All primary antibodies were diluted 1:1,000 in 5% bovine serum albumin (Bovogen Biologicals, Australia). The membranes were then incubated with the appropriate horseradish peroxidase-conjugated secondary antibodies (Cell Signaling Technology). Protein bands were visualized using an enhanced chemiluminescence detection system (NIPPON Genetics EUROPE, Germany) and quantified by densitometric analysis using ImageJ software (National Institutes of Health, USA). Protein expression levels were normalized to β-actin. For the western blot analysis, three experiments were performed independently (*n* = 3).

### Animal Model and Diet

Male C57BL/6 mice (6 weeks of age) were purchased from Daehan Bio Link (Republic of Korea). All animal procedures were approved by the Animal Ethics Committee of Cell Biotech (approval number CBT-2024-11) and were conducted in accordance with relevant institutional and national guidelines. After a 1-week acclimatization period, mice were randomly assigned to the following four groups (*n* = 7 per group): (1) normal chow diet (NCD + vehicle (PBS)); (2) HFD (60% of the total kilocalories derived from fat + vehicle); (3) BR3LP3-Low (HFD + 1.75 × 10^8^ CFU/day probiotic mixture); and (4) BR3LP3-High group (HFD + (1.75 × 10^9^ CFU/day probiotic mixture). The probiotic mixture contained BR3 and LP3 at a CFU ratio of 1:1. Obesity in the HFD, BR3LP3-Low, and BR3LP3-High groups was induced by feeding the mice an HFD for 5 weeks. After obesity induction, mice in the BR3LP3 treatment groups received daily oral administration of the probiotic mixture via oral gavage, whereas the HFD group received the vehicle only, for an additional 6 weeks.

Body weight was monitored weekly throughout the experimental period. For endpoint analysis, the final body weight was measured immediately before sacrifice at the end of the experimental period. Food intake was recorded twice weekly. At the end of the experiment, body compositions were analyzed using dual-energy X-ray absorptiometry (DEXA) scans (InAlyzer, Republic of Korea). The mice were then sacrificed after overnight fasting, and blood and tissue samples (liver and adipose tissues) were collected for further biochemical and molecular analyses.

### Body Composition Analysis

Whole-body fat and lean mass compositions were quantified via DEXA scans, while DEXA scan color-mapping images were obtained for qualitative visualization of body fat and lean mass composition.

### Histological Analysis

Liver tissue was dissected from the mice and fixed in 10% neutral-buffered formaldehyde (pH 6.8) overnight at room temperature. After fixation, the tissue was dehydrated using graded ethanol solutions, cleared in xylene, and embedded in paraffin. Subsequently, the samples were sectioned at 4 μm thickness using a microtome (Leica Microsystems, USA), deparaffinized in xylene, rehydrated through graded ethanol solutions (100% to 70%), and rinsed in distilled water. Liver sections were stained with hematoxylin and eosin to evaluate hepatic morphology and lipid droplet formation. Histological images were captured using a light microscope (Olympus BX53, Japan) under identical exposure conditions. Hepatic steatosis was quantified by counting the number of lipid droplets per field using ImageJ software. Quantification was performed in at least five randomly selected microscopic fields of liver tissue per animal at 400x magnification.

### Serum Biochemical Analysis

Blood samples were collected and centrifuged at 2,000 × g for 10 min to obtain serum. Although the animal study included 7 mice per group, sufficient blood volume could not be obtained from some animals during sample collection. Therefore, the number of samples used for serum biochemical and metabolic hormone analyses varied according to sample availability, and the sample sizes are indicated in each corresponding figure legend. Serum levels of aspartate aminotransferase (AST), alanine aminotransferase (ALT), high-density lipoprotein (HDL), low-density lipoprotein (LDL), and total cholesterol (TC) were measured using an XL-100 automated chemistry analyzer (Erba, Czech Republic). Serum triglyceride (TG) levels were determined using a commercial assay kit (Invitrogen, USA). Serum leptin, adiponectin, and insulin levels were measured using commercially available enzyme-linked immunosorbent assay (ELISA) kits following the manufacturers’ instructions, including the Mouse Leptin ELISA Kit (Invitrogen), Mouse Adiponectin ELISA Kit (ABclonal, USA), and Mouse Ultrasensitive Insulin ELISA (ALPCO, USA).

### Real-Time Quantitative Polymerized Chain Reaction (qPCR)

Total ribonucleic acid (RNA) was extracted from liver tissues using TRIzol reagent (Invitrogen), according to the manufacturer’s instructions. First-strand complementary (c) deoxyribonucleic acid (DNA) synthesis was performed with 1 μg of total RNA using the Prime ScriptII 1st Strand cDNA Synthesis Kit (TaKaRa Bio, Japan) by following the manufacturer’s protocol. qPCR was performed using SYBR Green (GENET Bio, Korea) on a CFX Opus 96 Real-Time PCR system (Bio-Rad, USA). Amplification was conducted under standard cycling conditions, according to the manufacturer’s recommendations. Gene expression levels were calculated using the 2^−ΔΔCt^ method relative to the glyceraldehyde 3-phosphate dehydrogenase gene (*Gapdh*) as the internal control. Each sample was analyzed in triplicate, and results were expressed as relative messenger (m)RNA expression normalized to the NCD group.

### Primer Sequences

The primer sequences used for gene expression analysis were as follows: *Gapdh* (forward, 5′-GCCTTCCGTGTTCCT ACC-3′; reverse, 5′-CTTCACCACCTTCTTGATGTC-3′), *Srebp1c* (forward, 5′-ATGCCATGGGCAAGT ACA CA-3′; reverse, 5′-AGATCTCTGCCAGTGTTG CC-3′), *Pparγ* (forward, 5′-CAAGAATACCAAAGTGCGATCAA-3′; reverse, 5′-GAG CTGGGTCTTTTCAGAATAATAAG-3′), *Cebpα* (forward, 5′-AGGTGCTGGAGTTGACCA GT-3′; reverse, 5′-CAGCCTAGAGATCCAGCGAC-3′), *Acc* (forward, 5′-GCGTCGGGTAGATCCAGTT-3′; reverse, 5′-CTCAGTGGGGCTTAGCTCTG-3′), and *Fas* (forward, 5′-GGAGGTGGTGATAGCCGGTAT-3′; reverse, 5′-TGGGTAATCCATAGAGCCCAG-3′).

### Statistics

Statistical analyses were performed using GraphPad Prism (GraphPad Software Inc., USA). Comparisons between two groups were conducted using unpaired Student’s *t*-tests, while multiple group comparisons were assessed via one-way analysis of variance (ANOVA) followed by Bonferroni’s post hoc test. Data herein are expressed as the mean ± standard error of the mean (SEM), and *p* < 0.05 is considered statistically significant.

## Results

### BR3LP3 Reduces OA-Induced Lipid Accumulation and Lipogenesis-Related Protein Expression in HepG2 Cells

**Fig. 1:**
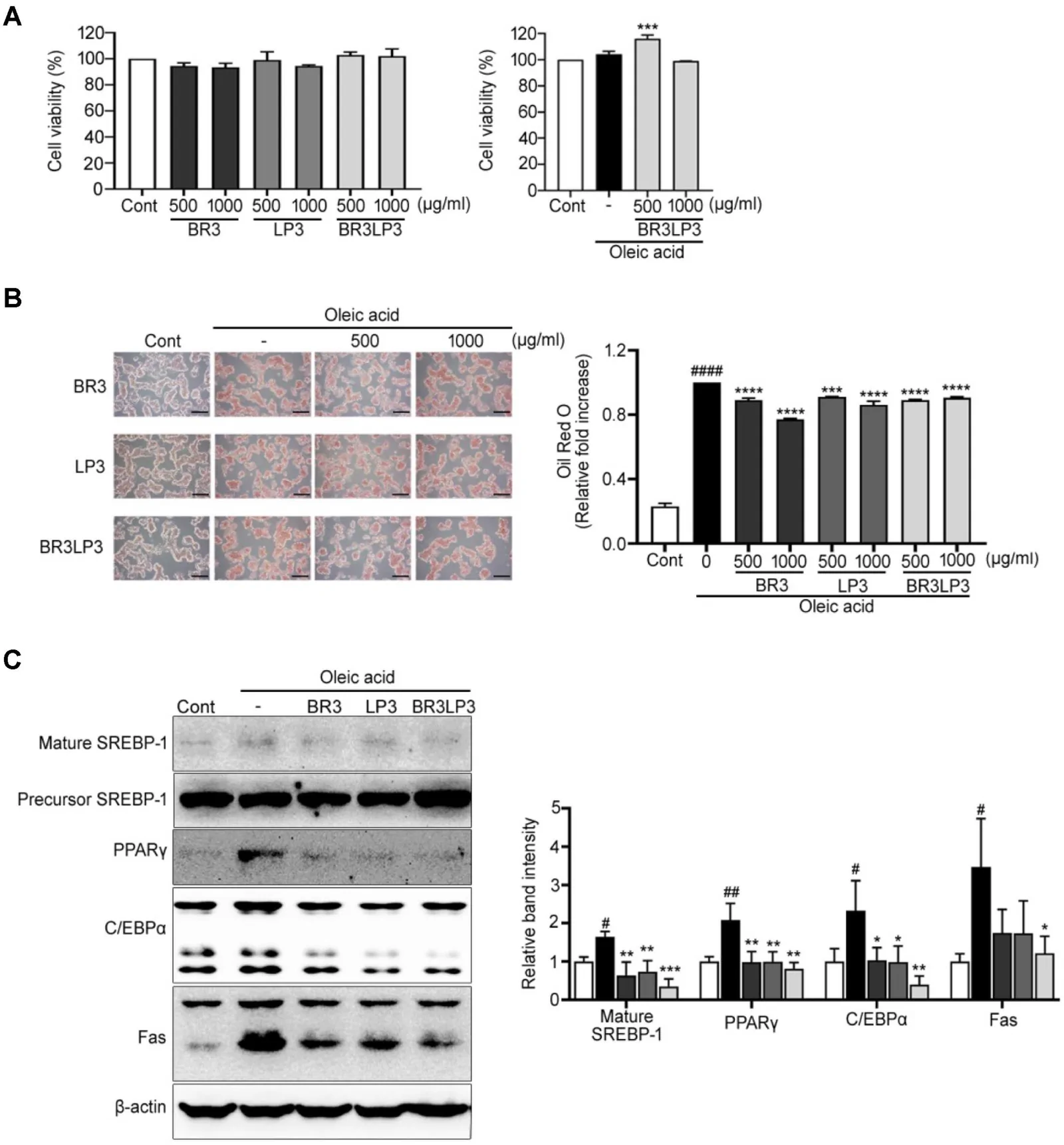
Effects of BR3LP3 Culture Supernatants on OA-Induced Steatosis in HepG2 Cells. (**A**) Cell viability of HepG2 cells treated with *Bifidobacterium breve* (BR3), *Lactiplantibacillus plantarum* (LP3), or their combination (BR3LP3) in the presence or absence of oleic acid (OA). (**B**) Representative Oil Red O staining images and quantitative analysis of intracellular lipid accumulation in OA-treated HepG2 cells following treatment with BR3, LP3, or BR3LP3 supernatant. (**C**) Protein expression levels of lipogenesis-related markers in HepG2 cells after treatment with OA and bacterial culture supernatants. Data are presented as the mean ± standard error of the mean (SEM) (*n* = 3 independent experiments). ^#^*p* < 0.05, ^##^*p* < 0.01, and ^####^*p* < 0.0001 versus the untreated control group; *p < 0.05, **p < 0.01, ***p < 0.001, and ****p < 0.0001 versus the OA-treated group. Cont, untreated control.

OA-induced steatosis was first established in HepG2 cells, which were then treated with BR3, LP3, or the BR3LP3 combination to examine the anti-steatotic potential of the latter. None of the treatments caused significant cytotoxicity in the absence or presence of OA (Fig. 1A), indicating that the observed effects were not caused by reducing cell viability. Oil Red O staining showed that after OA had markedly increased intracellular lipid accumulation, treatment with BR3, LP3, or BR3LP3 attenuated the OA-induced lipid deposition (Fig. 1B). Consistently, western blot analysis demonstrated that OA-induced expression of lipogenesis-related proteins, including SREBP-1, PPARγ, C/EBPα, and Fas, was reduced by treatment with the BR3, LP3, or BR3LP3 supernatant (Fig. 1C). Taken together, these findings indicate that BR3LP3 exerts an anti-steatotic effect on HepG2 cells without inducing cytotoxicity. Therefore, this combined formulation was selected for the subsequent *in vivo* study.

### BR3LP3 Attenuates HFD-Induced Metabolic Alterations and Hepatic Steatosis in Mice

**Fig. 2:**
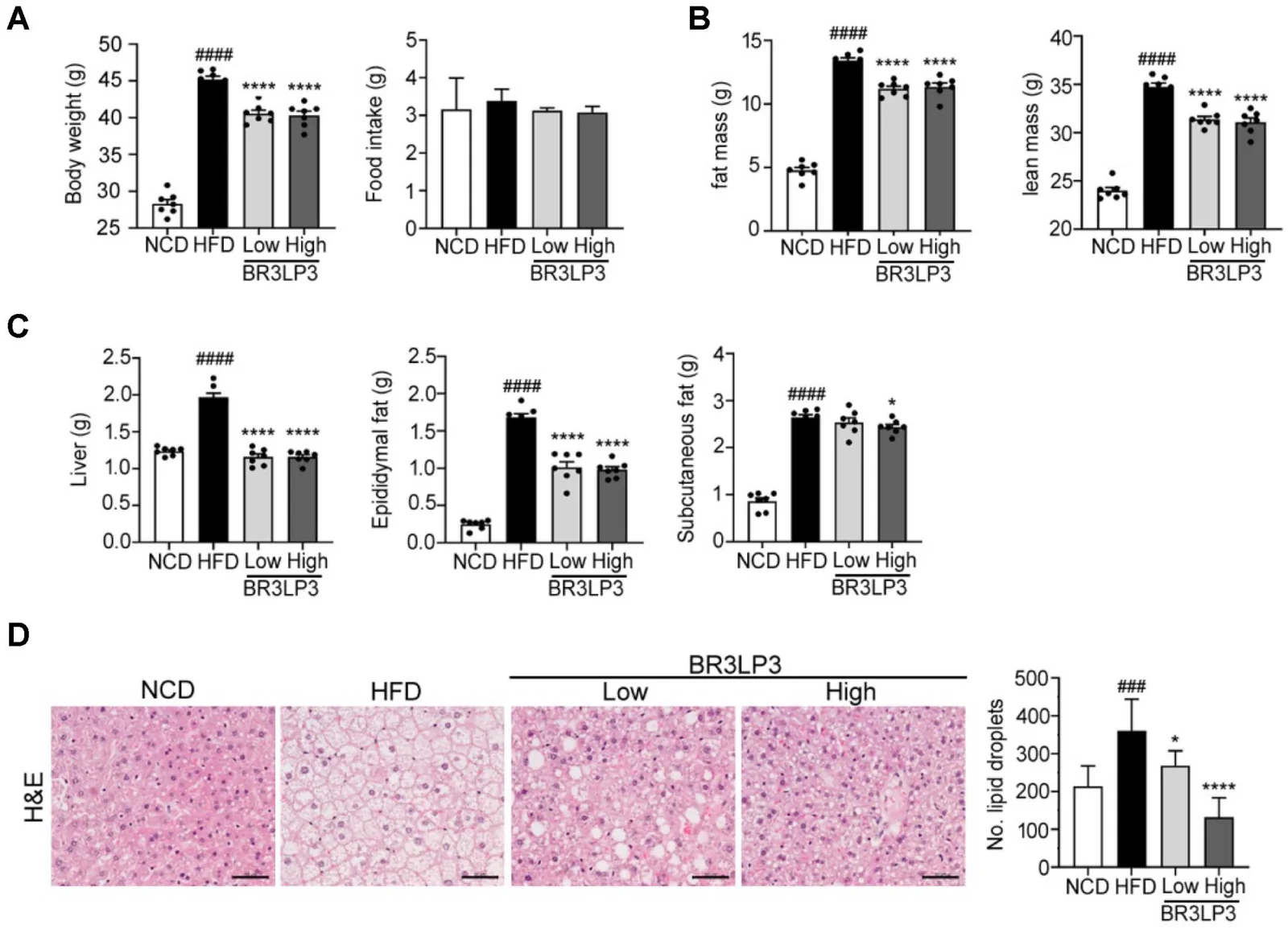
Effects of BR3LP3 on the Final Body Weight, Body Composition, Tissue Weight, and Hepatic Steatosis in HFD-Fed Mice. (**A**) Final body weight measured immediately before sacrifice and food intake in mice fed a normal chow diet (NCD), a high-fat diet (HFD), or an HFD supplemented with low-dose (1.75 × 10^8^ CFU/day) or high-dose (1.75 × 10^9^ CFU/day) BR3LP3. (**B**) Whole-body fat mass and lean mass measured by using dual-energy X-ray absorptiometry (DEXA). (**C**) Weights of livers, epididymal fat pads, and subcutaneous fat. (D) Representative hematoxylin and eosin (H&E)–stained liver sections and quantitative analysis of hepatic lipid droplet numbers. Scale bar = 50 μm. Data are presented as the mean ± SEM (*n* = 7). ^###^*p* < 0.001 and ^####^*p* < 0.0001 versus the NCD group; *p < 0.05 and ****p < 0.0001 versus the HFD group.

Obesity was first induced in mice by feeding them an HFD containing 60% of the total kilocalories derived from fat, after which the *in vivo* efficacy of BR3LP3 was evaluated. At the end of the experimental period, the final body weights measured immediately before sacrifice were significantly higher in the HFD group than in the NCD group (Fig. 2A). In contrast, both low- and high-dose BR3LP3 supplementation significantly reduced final body weights compared with those of the HFD group. Food intake did not differ significantly among the groups, indicating that the reduction in body weight observed in both BR3LP3 dose groups was not attributable to reduced food intake.

The body compositions of the mice measured via DEXA scans reveal that HFD feeding markedly increased the whole-body fat mass and lean mass compared with the NCD control group, whereas both low- and high-dose BR3LP3 supplementation significantly reduced the fat mass and lean mass compared with the HFD group (Fig. 2B). Consistently, liver, epididymal fat-pad, and subcutaneous fat tissue weights were significantly higher in HFD-fed mice than in the NCD control group (Fig. 2C). Although both low- and high-dose BR3LP3 supplementation significantly reduced liver weight and epididymal fat-pad weight compared with the HFD group, a significant reduction in subcutaneous fat weight was only observed in the high-dose group.

Histological analysis demonstrated pronounced lipid droplet accumulation in the livers of HFD-fed mice, whereas both low- and high-dose BR3LP3 supplementation reduced hepatic lipid accumulation (Fig. 2D). Meanwhile, quantitative analysis confirmed a significant reduction in hepatic lipid droplet numbers in both BR3LP3 dose groups compared with the HFD group. Collectively, our findings indicate that both low- and high-dose BR3LP3 supplementation attenuated HFD-induced increases in body weight, adiposity, and hepatic lipid accumulation.

### BR3LP3 Supplementation Improves Serum Lipid Profiles and Liver Injury Markers in HFD-Fed Mice

**Fig. 3:**
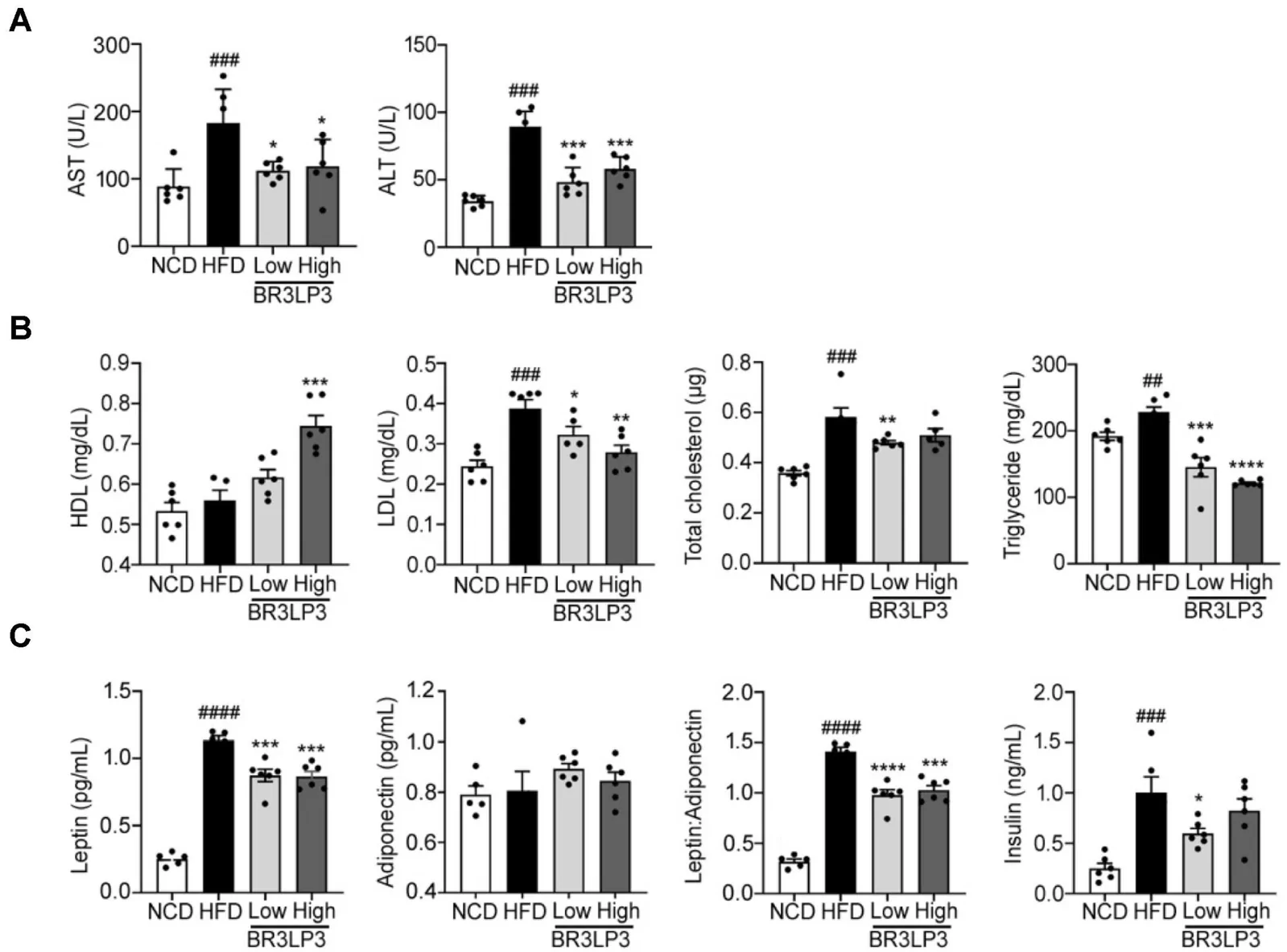
Effects of BR3LP3 on Serum Biochemical and Metabolic Parameters in HFD-Fed Mice. (**A**) Serum aspartate aminotransferase (AST) and alanine aminotransferase (ALT) levels (*n* = 6). (**B**) Serum lipid parameters high-density lipoprotein (HDL), low-density lipoprotein (LDL), total cholesterol, and triglyceride levels (*n* = 5 or 6 per group depending on serum availability). (**C**) Serum metabolic hormone-related parameters leptin, adiponectin, the leptin:adiponectin ratio, and insulin levels (*n* = 5 or 6 per group depending on serum availability). Data are presented as the mean ± SEM (*n* = 5 or 6). ##*p* < 0.01, ^###^*p* < 0.001, and ^####^*p* < 0.0001 versus the NCD group; *p < 0.05, **p < 0.01, ***p < 0.001, and ****p < 0.0001 versus the HFD group.

Serum biochemical parameters for lipid levels and liver injury were analyzed to evaluate the metabolic effects of BR3LP3. Feeding the HFD significantly elevated serum AST and ALT levels compared with the NCD group, thereby indicating hepatic injury caused by the HFD, while both low- and high-dose BR3LP3 supplementation significantly reduced AST and ALT levels relative to the HFD group (Fig. 3A). HFD-fed mice also showed dyslipidemia, characterized by increased LDL, TC, and TG levels (Fig. 3B). Although both BR3LP3 dose groups showed significant reductions in LDL and TG levels compared with the HFD group, TC was significantly reduced in the low-dose group only, whereas HDL was significantly increased in the high-dose group only.

Feeding the HFD significantly increased serum leptin and insulin levels and elevated the leptin:adiponectin ratio (Fig. 3C). While the adiponectin level did not markedly change, both low- and high-dose BR3LP3 supplementation significantly reduced the leptin level, and thus lowered the leptin:adiponectin ratio, compared with the HFD group. Serum insulin levels were significantly reduced in the low-dose BR3LP3 group only, whereas the high-dose group showed a similar but non-significant decrease. Taken together, these findings indicate that BR3LP3 favorably altered several serum metabolic parameters and liver injury-related markers in HFD-fed mice.

### BR3LP3 Reduces the Hepatic Expression of Lipogenesis-Related Genes in HFD-Fed Mice

**Fig. 4:**
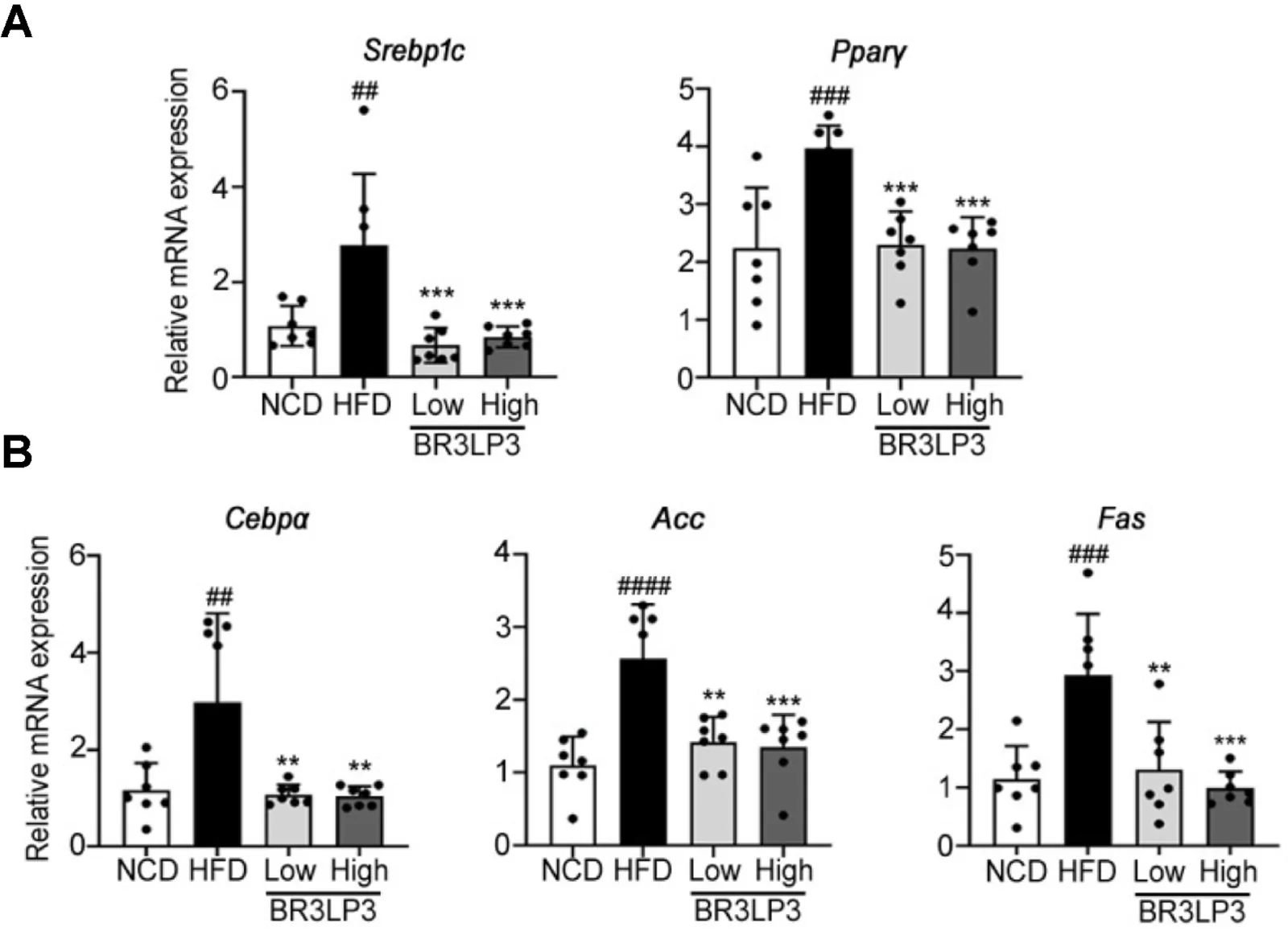
Hepatic Expression of Lipogenesis-Related Genes in BR3LP3-Treated HFD-Fed Mice. Relative messenger RNA (mRNA) expression levels for *Srebp1c*, *Pparγ*, *Cebpα*, *Acc*, and Fas in liver tissues. Gene expression was normalized to *Gapdh* and is expressed relative to the NCD group. Data are presented as the mean ± SEM (*n* = 7). ^##^*p* < 0.01, ^###^*p* < 0.001, and ^####^*p* < 0.0001 versus the NCD group; **p < 0.01 and ***p < 0.001 versus the HFD group.

To examine whether BR3LP3 was associated with changes in hepatic lipogenesis-related gene expression, we analyzed the mRNA levels of representative lipogenesis-related markers *Srebp1c*, *Pparγ*, *Cebpα*, *Acc*, and *Fas* in liver tissues using qPCR. HFD-fed mice had significantly upregulated mRNA expression of all of these markers compared with the NCD group (Fig. 4), while both low- and high-dose BR3LP3 supplementation significantly suppressed their HFD-induced upregulation. These findings indicate that the reduction in hepatic lipid accumulation following BR3LP3 supplementation was accompanied by decreased expression of these hepatic lipogenesis-related genes.

## Discussion

In this study, we found that BR3LP3 reduced lipid accumulation in OA-treated HepG2 cells and attenuated hepatic lipid accumulation in HFD-fed obese mice. In the latter experiment, BR3LP3 supplementation reduced body weight, whole-body fat mass, liver weight, and epididymal fat-pad weight and improved several serum metabolic parameters without affecting food intake. These changes were accompanied by decreased hepatic expression of lipogenesis-related genes. Collectively, the findings indicate that reduced hepatic lipid accumulation following BR3LP3 supplementation broadly improved adiposity and systemic metabolic status.

Hepatic steatosis is a key early event in metabolic liver disease and is closely associated with dysregulated lipid metabolism [17, 18]. In our study, BR3LP3 significantly reduced hepatic steatosis, as evidenced by the decreased liver weight and lipid accumulation in HFD-fed mice supplemented with the combined probiotic, which is consistent with the noted reduction in OA-induced lipid accumulation in HepG2 cells treated with the BR3LP3 supernatant. Our findings support the anti-steatotic potential of BR3LP3 both *in vitro* and *in vivo*, albeit the delivery route (BR3LP3 supernatant in the OA-induced lipid accumulation HepG2 experiment and oral administration of viable BR3LP3 to the mice) may represent biologically distinct interventions.

A major finding of this study was that the reduction in hepatic lipid accumulation was accompanied by decreased expression of lipogenesis-related markers. Dysregulated *de novo* lipogenesis contributes to hepatic lipid accumulation through transcriptional regulators such as SREBP-1c, PPARγ, and C/EBPα and their downstream enzymes such as ACC and FAS [18, 19]. We found that BR3LP3 treatment reduced lipogenesis-related protein expression in OA-treated HepG2 cells and decreased hepatic expression of *Srebp1c*, *Pparγ*, *Cebpα*, *Acc*, and *Fas* in HFD-fed mice. These findings support an association between BR3LP3 treatment and reduced expression of lipogenesis-related markers.

BR3LP3 supplementation also improved several systemic metabolic parameters; it reduced serum AST and ALT levels induced by the HFD (thereby indicating favorable changes in these liver injury-related biochemical markers) together with improvements in circulating lipid parameters. BR3LP3 supplementation also reduced serum leptin levels and the leptin-to-adiponectin ratio at both doses, whereas serum insulin was significantly reduced only in the low-dose group. These findings indicate that BR3LP3 supplementation reduced hepatic lipid accumulation alongside broadly improving systemic metabolic and endocrine status, including adipose tissue-derived hormonal signaling, although a direct hepatic contribution cannot be excluded. Importantly, these effects were observed without significant differences in food intake, suggesting that they cannot be attributed solely to reduced caloric intake. Nevertheless, because BR3LP3 supplementation reduced body weight and adiposity concurrently with hepatic lipid accumulation, we could not determine whether the hepatic improvement resulted from direct action on the liver or occurred secondarily due to improved systemic metabolic status. Reduced delivery of circulating fatty acids from adipose tissue to the liver, together with changes in metabolic hormone signaling, could have contributed to the observed reduction in hepatic lipid accumulation. Accordingly, the hepatic findings should be interpreted as part of a broad metabolic response rather than as evidence of an exclusively liver-specific effect.

BR3LP3 did not show a clear dose-dependent response within the tested range. Probiotic efficacy is generally strain- and outcome-specific, and it should not be assumed that an increase in administered CFU necessarily produces a proportional increase in efficacy [20]. Consistent with this principle, comparable responses between low and high doses have been reported for *Bifidobacterium animalis* subsp. *lactis* BB-12, for which a possible ceiling effect was proposed, and for *Lactobacillus gasseri* BNR17 in a diet-induced obesity model [21, 22]. In addition, a multi-dose study of *Lacticaseibacillus paracasei* K56 demonstrated more consistent body fat-related improvements with an intermediate dose than with the highest dose [23]. Thus, the low dose of BR3LP3 administered in the present study could have been sufficient to reach the biological response threshold under the present experimental conditions. However, because intestinal recovery, persistence, and bacterial-derived metabolites were not assessed, the biological basis for the apparent plateau cannot be clarified.

The present findings should also be interpreted in relation to previous BR3LP3 studies. Yun *et al*. [13] reported that BR3LP3 ameliorated HFD-induced obesity and was associated with reductions in body weight, adiposity, serum transaminase levels, and hepatic lipid accumulation, together with changes in adipose tissue lipid metabolism, intestinal barrier-related markers, and gut microbiota composition. Thus, several *in vivo* outcomes in the present study overlap with those earlier observations. The incremental contribution of the present study is the more focused characterization of hepatic lipid accumulation through employing an OA-induced HepG2 model and targeted analyses of lipogenesis-related proteins in hepatocyte-like cells and lipogenesis-related genes in liver tissue in the mouse model. Hence, our findings extend those from the previous study by showing that reduced hepatic lipid accumulation is accompanied by changes in hepatic lipogenesis-related markers, although they do not establish a distinct liver-specific or causal mechanism.

Metabolic effects consistent with our observations have also been reported in overweight dogs, in which BR3LP3 supplementation reduced body weight-related parameters, serum triglyceride, TC, leptin, and insulin levels without affecting food intake or physical activity [14]; BR3LP3 supplementation also altered their gut microbial composition, suggesting that modulation of the intestinal environment contributes to its broad metabolic effects. However, the gut–liver axis was not directly examined in the present study, and its involvement therefore remains hypothetical. Taken together, the available evidence supports further investigation of BR3LP3 as a potential probiotic strategy for hepatic steatosis and related metabolic abnormalities. However, additional mechanistic and human intervention studies are required before clinical efficacy can be established.

### Limitation

This study has several limitations. First, due to the experimental design, we could not distinguish whether the reduction in hepatic lipid accumulation is directly mediated within the liver or occurs secondarily to reductions in body weight, adiposity, and systemic metabolic abnormalities. Pair-fed or body weight-matched controls and liver-specific mechanistic approaches are required to distinguish these possibilities.

Second, although we could associate BR3LP3 with the reduced hepatic expression of lipogenesis-related genes, hepatic protein expression, enzyme activity, and lipid flux were not assessed. Therefore, a further study is needed to identify the causal mechanism underlying the suppression of hepatic lipogenesis.

Third, inflammatory activity, hepatocyte ballooning, fibrosis, and standardized steatohepatitis scores were not evaluated in the present study. Therefore, a further study incorporating these endpoints is needed to determine whether the effects observed in hepatic steatosis can be extended to steatohepatitis or other more advanced stages of liver disease.

Finally, the potential involvement of the gut–liver axis was not directly examined. Future studies should evaluate gut microbiota composition, microbial metabolites, intestinal barrier function, and inflammatory signaling.

## Conclusion

In conclusion, BR3LP3 reduces lipid accumulation in OA-treated HepG2 cells and attenuates hepatic steatosis in HFD-fed mice. *In vivo*, these changes were accompanied by reductions in body weight and adiposity, improvements in systemic metabolic parameters, and decreased expression of hepatic lipogenesis-related genes. Although the present study does not distinguish direct hepatic effects from secondary effects associated with improved systemic metabolism, the findings indicate that BR3LP3 supplementation shows potential for development as a probiotic strategy targeting hepatic steatosis and related metabolic abnormalities.
